# Late-Onset Chylothorax After Coronary Artery Bypass Grafting

**DOI:** 10.7759/cureus.48843

**Published:** 2023-11-15

**Authors:** Bogdan Okiljević, Marko Nemet, Ranko Zdravkovic, Sanja Ergelašev, Ivan Ergelašev

**Affiliations:** 1 Clinic for Cardiovascular Surgery, Institute for Cardiovascular Diseases Dedinje, Beograd, SRB; 2 Internal Medicine, Faculty of Medicine, University of Novi Sad, Novi Sad, SRB; 3 Clinic for Cardiovascular Surgery, Institute for Cardiovascular Diseases of Vojvodina, Sremska Kamenica, SRB; 4 Clinic for Cardiology, Institute for Cardiovascular Diseases of Vojvodina, Sremska Kamenica, SRB; 5 Clinic for Thoracic Surgery, Institute for Pulmonary Diseases of Vojvodina, Sremska Kamenica, SRB

**Keywords:** coronary artery bypass grafting (cabg), pleural effusion, talc pleurodesis, chest tube drainage, chylothorax

## Abstract

Chylothorax, the presence of lymph in the pleural cavity, is a significant post-cardiac surgery complication. Historically linked to left internal mammary artery (LIMA) harvesting, its occurrence in cases without LIMA usage is uncommon. This paper details a case of chylothorax in an 84-year-old female patient who underwent coronary artery bypass grafting (CABG) without LIMA harvesting. Three months post-surgery, she manifested symptoms of exertional shortness of breath and diminished breath sounds on the left side. Diagnostic measures, including echocardiography and chest X-rays, revealed a pronounced left-sided pleural effusion. Diagnostic thoracocentesis yielded a milky fluid, and laboratory analysis confirmed its chylous nature. Therapeutic interventions comprised chest tube insertion, drainage of the milky fluid, dietary modifications, and the performance of talc pleurodesis after a fatty food-provocation test resulted in increased fluid collection. The patient's journey highlights the challenges of diagnosing and managing post-cardiosurgical chylothorax. The paper emphasizes the importance of early detection and appropriate interventions to prevent complications associated with a heightened mortality risk.

## Introduction

Chylothorax refers to the presence of lymph within the pleural cavity. Even though it is a rare complication of cardiac surgery, it poses a potentially serious risk, with fewer than 50 cases described in English literature [1]. This condition is more prevalent in the pediatric population post-cardiac surgery than in adult patients [2]. Many of the documented cases of chylothorax after cardiac surgery are linked to left internal mammary artery (LIMA) harvesting, with most cases manifesting between the second and tenth postoperative days (POD) [3,4].

Here, we present a patient who developed left-sided chylothorax three months after undergoing coronary artery bypass grafting (CABG) without LIMA harvesting. The patient received treatment at both the Institute for Pulmonary Diseases of Vojvodina and the Institute for Cardiovascular Diseases, Dedinje. The Institute for Pulmonary Diseases of Vojvodina is a leading tertiary healthcare institution, providing care to a vast population in Vojvodina, while the Institute for Cardiovascular Diseases, Dedinje, stands as a hallmark for advanced cardiac care in the Republic of Serbia. This case provides insights into the diagnosis, clinical progression, and therapeutic approach for managing post-CABG chylothorax.

## Case presentation

An 84-year-old female patient with a history of chronic hypertension presented with symptoms of exertional shortness of breath and orthopnea. During her initial visit, an echocardiogram revealed abnormalities in the motion of the left ventricular wall. Subsequent coronary angiography showed multivessel coronary artery disease: 35% stenosis in the proximal left anterior descending artery (LAD), 75% stenosis in the proximal left circumflex artery (LCx), 75% stenosis in the obtuse marginal 2 (OM2) artery, and 98% stenosis in the proximal right coronary artery (RCA). After failing to respond to conservative treatment, including medication and a cardiac rehabilitation program, the patient was referred to a cardiac surgeon who recommended a CABG procedure. The patient underwent an elective CABG using saphenous vein grafts. The LIMA was not harvested due to the patient's advanced age and suboptimal preoperative condition. Consequently, the decision to avoid using LIMA was aimed at reducing operative time and facilitating a faster postoperative recovery. Following a smooth postoperative period, the patient was successfully extubated on the day of surgery, transferred to the ward on the first postoperative day (POD 1), and had pericardial catheters removed on POD 2. The patient was then discharged home on POD 7. A postoperative chest X-ray (CXR) showed no signs of pleural effusion. During the first follow-up appointment six weeks after the procedure, the patient reported no complaints, and a CXR was not performed.

**Figure 1:**
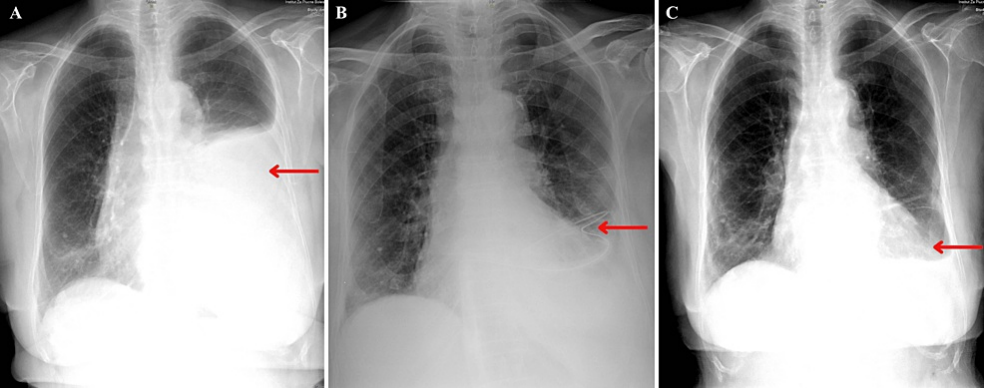
Three anterior-posterior view chest X-rays of the patient with chylothorax (A) Initial chest X-ray; red arrow shows a large, left-sided pleural effusion. (B) Chest X-ray following the insertion of a left-sided chest tube; red arrow shows the chest tube and a partial resolution of the pleural effusion. (C) Chest X-ray following evacuation of a left-sided chest tube and left-sided talc pleurodesis; red arrow shows the complete resolution of the pleural effusion.

Three months post-surgery, the patient presented with exertional shortness of breath and muffled breath sounds upon auscultation of the left hemithorax. These symptoms led to further investigations with electrocardiography and echocardiography, both of which were within normal limits, except for a left-sided pleural effusion identified on the echocardiography scan. The CXR confirmed a large left-sided pleural effusion (Figure 1A). The patient was hospitalized, and a left-sided diagnostic thoracocentesis produced a milky-appearing fluid. Laboratory examination of the pleural fluid indicated a sterile composition consistent with chylothorax, evidenced by its high lipid content (triglycerides 341.27 mg/dl and cholesterol 44.33 mg/dl) and a low count of mixed cells. Chest tubes were inserted, and 1600 mL of milky-appearing fluid was drained. Subsequent CXR showed a significantly reduced left-sided pleural effusion (Figure 1B). In the following hospital days, a low-fat diet was initiated, and pleural drainage ceased. A fatty food-provocation test led to an increase in chest tube output to 350 mL per day. To prevent fluid reaccumulation, talc pleurodesis was performed. After the procedure, the output from the chest tubes significantly decreased and stopped entirely after three days. The chest tube was removed on the tenth day of hospitalization. A follow-up CXR showed no fluid reaccumulation (Figure 1C), and the patient was discharged home 12 days after initial hospitalization.

## Discussion

Post-cardiosurgery chylothorax can result in a range of significant complications. These include nutritional deficiencies, dehydration, and immunosuppression, stemming from the loss of lymphocyte- and lipid-rich fluid into the pleural space. Additionally, respiratory failure may arise due to the external restriction on the expansion of lung parenchyma. The fatty fluid in the pleural space also creates an ideal medium for bacterial growth, increasing the risk of infections like empyema. Such complications not only extend hospital stays but also escalate treatment costs [4,5]. If not managed appropriately, the mortality rate can soar to as high as 50% [3].

The primary mechanism leading to chylothorax is the harvesting of LIMA and the associated damage to the left anterior mediastinal lymph node chain [4,6]. In our patient's case, LIMA was not harvested. The chylothorax likely developed due to disruption of anomalous peri-thymic lymphatics during the procedure, resulting in a "low-output" lymph secretion into the left pleural space. According to our institutional guidelines, we routinely remove residual thymic tissue located beneath the left brachiocephalic vein during every CABG procedure. We believe that this practice is the most probable cause of chylothorax, likely due to the injury of an aberrant thoracic duct that flows into the brachiocephalic vein. This variant anatomy is well-documented in literature [7]. Another potential cause could be the presence of multiple terminal branches of the thoracic duct near the left brachiocephalic vein, which could be easily damaged during the removal of thymic tissue. Additionally, left-sided pericardial stay sutures were placed, but this was deemed highly unlikely to be the cause of chylothorax. Notably, the left pleural space was neither invaded nor injured during the procedure. Due to the slow development and minimal lymph leakage, the patient did not exhibit metabolic, nutritional, or immunological disturbances. Her symptoms were predominantly respiratory.

In patients with chylothorax, both physical examination and imaging typically reveal nonspecific pleural effusion. The pleural fluid's appearance can range from milky or serous to serosanguineous or bloody. A high triglyceride level in the fluid is diagnostic of chylothorax, with a sensitivity of 99% [8].

**Figure 2:**
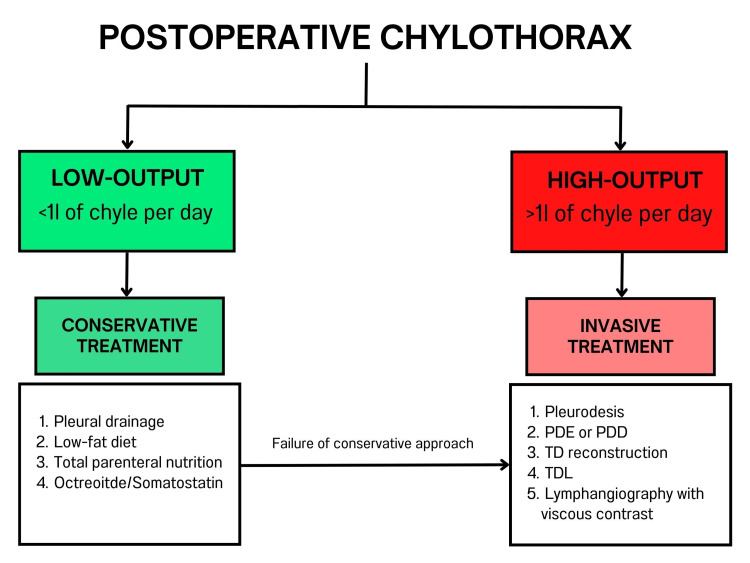
Proposed management strategies for postoperative chylothorax This figure presents the proposed treatment protocol for postoperative chylothorax, categorized by chyle output levels. For low-output chylothorax, defined as less than 1 liter of chyle per day, conservative treatments are initially recommended. Should these conservative measures fail, more invasive treatments are considered. Conversely, for high-output chylothorax, which involves more than 1 liter of chyle per day, invasive treatments are advised as the first line of action [9,10]. Abbreviations: PDE - percutaneous duct embolization; PDD - percutaneous duct disruption; TD - thoracic duct; TDL - thoracic duct ligation

The low incidence rate of chylothorax following cardiac surgery, combined with the scarcity of relevant studies, has posed significant challenges in developing preventive and therapeutic measures for post-surgical chylothorax. The primary aim of treating post-surgical chylothorax is to evacuate the accumulated lymph from the pleural space, prevent its reaccumulation, and further lymph loss. Proper management of chylothorax substantially reduces the risks of all the aforementioned complications. Treatment can be divided into conservative and surgical approaches (Figure 2).

Conservative management of chylothorax typically includes several strategies: dietary modifications, total parenteral nutrition, subcutaneous octreotide infusion, pleural drainage, and pleurodesis [11]. For patients with low-output chylothorax, the initial treatment usually combines dietary modifications with pleural drainage. The cornerstone of dietary modification is the reduction of fat intake, particularly long-chain fatty acids (LCF). By substituting LCF with medium-chain fatty acids (MCF), which are absorbed directly into the bloodstream and bypass the chylomicron-chyle duct system, there is a notable decrease in the production and accumulation of chyle in the pleural space [12,13].

Surgical intervention is indicated when conservative therapy fails to manage chylothorax effectively. The preferred surgical approach is video-assisted thoracic duct ligation (TDL) [14]. However, in certain complex cases, alternative procedures may be necessary. These can include transabdominal or transthoracic duct reconstruction, percutaneous thoracic duct embolization or disruption (TDE/TDD), lymphangiography using highly viscous Lipiodol, or even the creation of a pleuroperitoneal shunt. It's important to note that these alternative surgical options are associated with higher risks of morbidity and mortality [15].

## Conclusions

Chylothorax following cardiac surgery, while a rare occurrence, can pose significant clinical challenges and potentially severe complications if not addressed promptly. The presented case highlights the unusual development of chylothorax in the absence of LIMA harvesting, suggesting that even routine cardiac procedures can lead to unexpected disruptions of the thoracic lymphatic system. The successful management of this patient underscores the importance of an early and accurate diagnosis, followed by tailored treatment strategies. While conservative management remains the first-line treatment for chylothorax, physicians should remain vigilant and consider surgical interventions when necessary.
